# Treatment of antibiotic-resistant bacteria colonizing diabetic foot ulcers by OLED induced antimicrobial photodynamic therapy

**DOI:** 10.1038/s41598-023-39363-4

**Published:** 2023-08-28

**Authors:** Marta Piksa, Wojciech Fortuna, Cheng Lian, Małgorzata Gacka, Ifor D. W. Samuel, Katarzyna Matczyszyn, Krzysztof J. Pawlik

**Affiliations:** 1 Ludwik Hirszfeld Institute of Immunology and Experimental Therapy, Polish Academy of Sciences, Weigla 12, 53-114 Wroclaw, Poland; 2 Department of Neurosurgery, Wroclaw Medical University, Borowska 213, 50-556 Wroclaw, Poland; 3 Organic Semiconductor Centre, School of Physics and Astronomy, SUPA, University of St Andrews, St Andrews, KY16 9SS UK; 4 DOBRZYŃSKA Medical Center, Wroclaw, Poland; 5 Advanced Materials Engineering and Modelling Group, Faculty of Chemistry, Wroclaw University of Science and Technology, Wyb. Wyspianskiego 27, 50-370 Wroclaw, Poland

**Keywords:** Antimicrobial resistance, Organic LEDs

## Abstract

We evaluate the efficacy of antimicrobial Photodynamic Therapy (APDT) for inactivating a variety of antibiotic-resistant clinical strains from diabetic foot ulcers. Here we are focused on APDT based on organic light-emitting diodes (OLED). The wound swabs from ten patients diagnosed with diabetic foot ulcers were collected and 32 clinical strains comprising 22 bacterial species were obtained. The isolated strains were identified with the use of mass spectrometry coupled with a protein profile database and tested for antibiotic susceptibility. 74% of isolated bacterial strains exhibited adaptive antibiotic resistance to at least one antibiotic. All strains were subjected to the APDT procedure using an OLED as a light source and 16 µM methylene blue as a photosensitizer. APDT using the OLED led to a large reduction in all cases. For pathogenic bacteria, the reduction ranged from 1.1-log to > 8 log (*Klebsiella aerogenes*, *Enterobacter cloaca*, *Staphylococcus hominis*) even for high antibiotic resistance (MRSA 5-log reduction). Opportunistic bacteria showed a range from 0.4-log reduction for *Citrobacter koseri* to > 8 log reduction for *Kocuria rhizophila*. These results show that OLED-driven APDT is effective against pathogens and opportunistic bacteria regardless of drug resistance.

## Introduction

According to the *Diabetes Atlas 10th edition* published by the International Diabetes Federation in 2021, there are 537 million people between 20 and 79 years old around the globe diagnosed with diabetes^1^. The worldwide prevalence of both type 1 and type 2 diabetes is continuously increasing and is forecast to reach 643 million people in 2030, and 783 million in 2045^2^.

A very serious complication of diabetes is a “diabetic foot”, defined as an ulcer in the lower limb as a result of peripheral artery disease and/or neuropathy caused by diabetes^3^. It poses a favorable environment for bacteria growth resulting in chronic infections which may reach bones^4^. Infections caused by antibiotic-resistant bacteria require supporting or emergency medical treatment. Growing and dynamically evolving antibiotic resistance among bacteria is considered one of the biggest global threats to public health and food security, entailing simultaneously huge economic costs associated with hospitalization^5^. Despite the fact that "superbugs" pose a threat to everyone, there are some groups at significantly higher risk including transplant recipients, people living with HIV, patients receiving chemotherapy, and those with primary immunodeficiency disorders or with neurological issues related to diabetes^6–8^.

The main method to save the infected limb is a medical revascularization or surgical reconstruction in ischemic diabetic foot assisted with antibiotics but if it fails or proves inadvisable for the patient, the only option is major amputation, 24-fold more frequent among people with diabetic foot in comparison to non-traumatic amputations^9^. Current estimates indicate that globally above 15% of diabetics are diagnosed with diabetic foot, and every year more than 1 million patients lose limbs as a consequence of diabetes. An amputation due to diabetes occurs every 30 s worldwide^10^. The mortality rate is in this case higher than for colon, prostate or breast cancer and it might achieve 55% when the diabetic foot develops or even 74% after amputation^11^.

**Figure 1:**
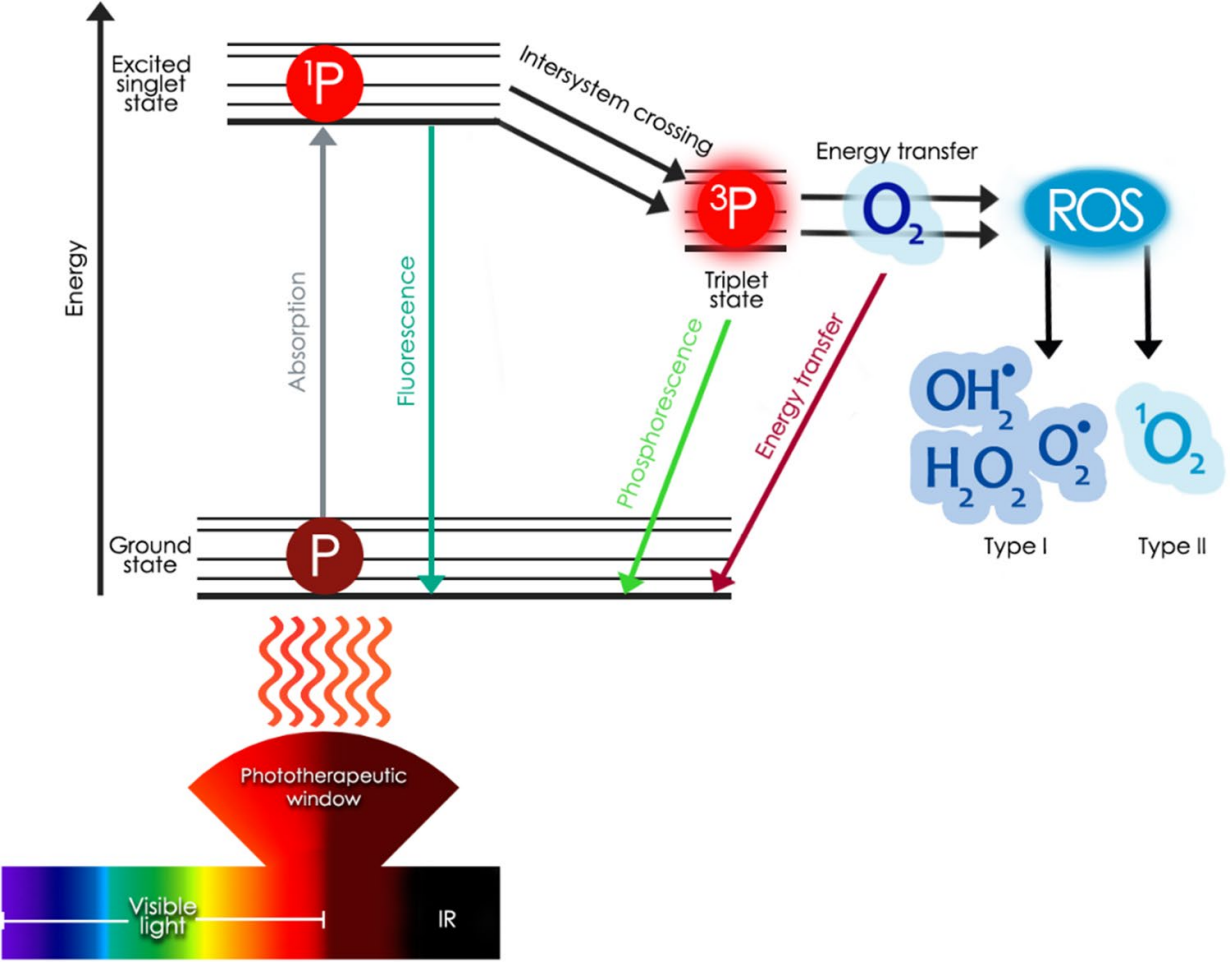
The mechanism of photodynamic action. Absorption of light energy by a photosensitizer leads to the generation of reactive oxygen species (ROS) considered as main antimicrobial agent.

Photodynamic therapy (PDT) is a promising light driven treatment, best known as an anticancer therapy. Is already medically approved and presented by the American Cancer Society as a type of radiation therapy. However, in contrast to radiotherapy, PDT uses visible light, so there is no ionizing radiation, and it may be applied many times at the same place. In addition to light, PDT involves a light-activated photosensitizer (PS) and molecular oxygen^12^. The excitation of the PS by light leads to intramolecular non-radiative energy transfers and in the end physical energy transfer to oxygen in the ground state (^3^O_2_^3^Ʃg^−^) resulting in the production of reactive oxygen species (ROS), mainly superoxide anion radical (${\text{O}}_{2}^{ \cdot - }$) and hydroperoxide radical (OH_2_^⋅^) (Type 1 of photodynamic actions) or singlet oxygen (^1^O_2_^1^∆_g_) formation (photochemical pathway Type 2) (Fig. 1)^13^. Both above pathways require molecular oxygen and lead to the oxidation of biomolecules, including proteins, lipids, and nitrogenous bases of nucleotides which has the effect of the destruction of pathogens and undesired cells^14^. According to current knowledge, the mechanism of PDT does not seem to remarkably depend on the type of cell (prokaryotic/eukaryotic). In contrast to normal human tissue, abnormal cells are characterized by higher sensitivity to oxidative stress caused by PDT, and the treatment itself is considered to not have long-term side effects^15^. The photodynamic action can be effective against a wide range of pathogens, notably viruses, bacteria, fungi, and parasites, starting a new chapter in antimicrobial treatment—antimicrobial photodynamic therapy (APDT)^16–19^. Through 22 years of PDT clinical administration, only 55 PDT incidents were reported^20,21^.

The conventional approach to PDT has used large and expensive light sources such as lamps, lasers, and large LED arrays; however, we propose the use of an OLED as a flexible, homogenous, and large surface light source. It allows for better control of the irradiation area and the delivered energy dose. In prior work, we have shown the potential of OLEDs for outpatient treatment of non-melanoma skin cancer^22^ and the in vitro efficacy of APDT using OLEDs in the elimination of *Staphylococcus aureus*^23^.

In the present work we explore the effectiveness of OLED based APDT on clinical pathogenic bacterial strains. We collected samples from patients with diabetic ulcers, and identified the bacteria and their antibiotic resistance. Then we proceeded to apply OLED to in vitro APDT.

## Results

The research is based on thirty-two bacterial strains and twenty-two bacterial species isolated from ten patients diagnosed with diabetic foot ulcers. Only aerobic bacteria were cultured and identified. For bacterial isolation, four different microbiological growth media were applied, including MacConkey agar, blood agar, BHI agar, and LB agar to overcome, at least partially, species selectivity. The best efficiency exhibited blood agar, with the highest number of colony-forming unit (CFU) and the greatest diversity of bacterial species. Obtaining pure bacterial cultures allowed the analysis of species with the use of MALDI Biotyper system. The identified strains have been deposited with the Polish Collection of Microorganisms (PCM).

The list of bacteria identified in the swabs from each patient is provided in Table 1. Between 2 and 4 bacterial species were isolated from each clinical swab. Seven bacterial species (*Corynebacterium striatum*, *Enterobacter cloacae*, *Proteus mirabilis*, *Proteus penneri*, *Staphylococcus aureus*, *Staphylococcus caprae* and *Streptococcus agalactiae*) appeared independently in more than one medical specimen (Table 1). The vast majority of bacterial species consist of staphylococci characteristic of bacterial flora of the human skin and skin infections. The most frequent *Staphylococcus *ssp. was *Staphylococcus aureus* (three specimens, one of them was MRSA, the rest MSSA) isolated independently from three different patients. Another frequently isolated gram-positive bacterial species was *Corynebacterium striatum* identified in three unrelated clinical samples*.* Among gram-negative bacteria *Enterobacter cloacae* and *Proteus mirabilis* were the most common, each isolated separately from three distinct medical specimens.

**Table 1:** Bacterial species isolated from patients diagnosed with diabetic foot and their PCM numbers.

Patient number	Bacterial species	PCM number
*1*	*Staphylococcus hominis*, CoNS, MSCNS, MLSB (inducible)	PCM 3060
	*Streptococcus agalactiae*, MLSB (constitutive)	PCM 3050
	*Staphylococcus haemolyticus*, CoNS MRCNS, MLSB (constitutive)	PCM 3061
	*Staphylococcus pasteuri,* CoNS MSCNS	PCM 3062
*2*	*Serratia liquefaciens*	PCM 3049
	*Pseudomonas monteilii*	PCM 3069
	*Proteus mirabilis*	PCM 3052
*3*	*Staphylococcus aureus*, MRSA, MLSB (constitutive)	PCM 3047
	*Staphylococcus caprae*, CoNS MSCNS	PCM 3063
*4*	*Pseudomonas aeruginosa*	PCM 3070
	*Proteus mirabilis*	PCM 3053
*5*	*Staphylococcus capitis*, CoNS MSCNS	PCM 3064
	*Staphylococcus epidermidis*, CoNS MSCNS	PCM 3068
	*Klebsiella aerogenes*	PCM 3056
	*Enterobacter cloacae*	PCM 3057
*6*	*Kocuria rhizophila*	PCM 3065
	*Enterobacter cloacae*	PCM 3058
	*Streptococcus agalactiae*, MLSB (constitutive)	PCM 3051
*7*	*Staphylococcus aureus*, MSSA	PCM 3048
	*Enterobacter cloacae*	PCM 3059
	*Staphylococcus lugdunensis*, MSCNS	PCM 3188
*8*	*Proteus penneri*	PCM 3055
	*Staphylococcus simulans*, MSCNS	PCM 3187
	*Citrobacter koseri*	PCM 3189
	*Staphylococcus caprae*, CoNS MSCNS, MLSB (constitutive)	PCM 3185
*9*	*Proteus mirabilis*	PCM 3054
	*Enterococcus faecalis*, HLSR	PCM 3066
	*Corynebacterium striatum*	PCM 3067
	*Proteus penneri*	PCM 3191
*10*	*Corynebacterium striatum*	PCM 3190
	*Staphylococcus cohnii*, MSCNS	PCM 3186
	*Staphylococcus aureus*, MSSA	PCM 3184

The number of bacterial strains identified in clinical swabs from one patient varies from two to four. Seven of the twenty-two species were isolated more than once. The identical composition of the bacterial microbiome was never obtained twice. *CoNS* Coagulase-negative staphylococci, *MLSB* resistance to macrolide, lincosamide and streptogramin B, *MSSA* methicillin-sensitive *Staphylococcus aureus*; *MSCNS* methicyllin-resistant coagulase-negative *Staphylococcus*; *MRSA* Methicillin-resistant *Staphylococcus aureus*, *HLSR* high-level streptomycin resistance.

Antibiotic susceptibility testing (AST) was performed to identify adaptive resistance to antibiotics and the results are shown in Table 2. In the thirty-one bacterial strains isolated *Kocuria rhizophila* was the only not-tested bacteria due to the lack of confirmed laboratory procedures. Twenty-three bacteria (74%) showed adaptive antibiotic resistance to at least one antibiotic. The vast majority were resistant to ciprofloxacin (29%), further amoxicillin (22.5%), and clavulanic acid (22.5%). The most resistant isolated species is methicillin-resistant *Staphylococcus aureus* (MRSA)*. Enterococcus faecalis* is the only bacteria with natural resistance to aminoglycosides, cephalosporins, and clindamycin but the identified phenotype is characterized by a high level streptomycin resistance (HLSR). Moreover, in five strains the constitutive mechanism was identified ensuring resistance to macrolides, lincosamides, and streptogramins B (MLSB) resistance. Twenty-one (68%) bacteria showed susceptibility to increased exposure to antibiotic, and fourteen strains (45%) showed both resistance and susceptibility to increased exposure (Table 2).

**Table 2:** Effect of APDT, antibiotic resistance and susceptibility to increased exposure to antibiotics among bacterial strains isolated from patients diagnosed with diabetic foot ulcers.

No.	Bacterial species (PCM numer)	Antibiotic resistance	Susceptible, increased exposure	Effect of APDT (reduction log)
1	*Staphylococcus aureus* (3047) MRSA, MLSB (constitutive)	Amikacin, carbapenems, cephalosporins, cipro*, levo**, clindamycin, cloxacillin, erythromycin, lincosamides, macrolides, penicillins, streptogramins b, tobramycin	–	5
2	*S. aureus* (3048) MSSA	–	Cipro*, levo**	No data
3	*S. aureus* (3084) MSSA	Amikacin, cipro*, levo**, tobramycin	–	No data
4	*S. capitis* (3064) CoNS MSCNS	–	Cipro*, levo**	5.2
5	*S. caprae* (3063) CoNS MSCNS	Amikacin, gentamicin, tobramycin	Cipro*, levo**	3
6	*S. caprae* (3185) CoNS MSCNS, MLSB (constitutive)	Amikacin, clindamycin, erythromycin, macrolides, gentamicin, lincosamides, streptogramins b, tobramycin	Cipro*, levo**	No data
7	*S. cohnii* (3186) MSCNS	–	Cipro*, levo**	2.9
8	*S. epidermidis* (3068) CoNS MSCNS	–	Cipro*, levo**	5.4
9	*S. haemolyticus* (3061) CoNS MRCNS, MLSB (constitutive)	Carbapenems, cephalosporins, clindamycin, erythromycin, methicillin, penicillins, tetracycline	Cipro*, levo**	2.6
10	*S. hominis* (3060) CoNS MSCNS, MLSB (inducible)	Amikacin, clindamycin, erythromycin, tetracycline, tobramycin	Cipro*, levo**	> 8
11	*S. pasteuri* (3062) CoNS MSCNS	Erythromycin, tetracycline	Cipro*, levo**	6.6
12	*S. simulans* (3187) MSCNS	–	Cipro*, levo**	4.4
13	*S. lugdunensis* (3188) MSCNS	–	Cipro*, levo**	4.6
14	*S. liquefaciens* (3049)	Amoxicillin/clavulanic acid	–	1
15	*S. agalactiae* (3050) MLSB (constitutive)	Erythromycin, clindamycin, lincosamides, macrolides, streptogramins b, tetracycline	Levo*	5.4
16	*S. agalactiae* (3051) MLSB (constitutive)	Erythromycin, clindamycin, lincosamides, macrolides, streptogramins b, tetracycline	Levo*	No data
17	*E. cloacae* (3057)	Amoxicillin/clavulanic acid, cipro*	Levo**	> 8
18	*E. cloacae* (3058)	Amoxicillin/clavulanic acid		No data
19	*E. cloacae* (3059)	Amoxicillin/clavulanic acid		No data
20	*K. aerogenes* (3056)	Cefotaxime, fosfomycin i.v		> 8
21	*E. faecalis* (3066) HLSR	Aminoglycosides, cephalosporins, clindamycin, streptomycin (high level; synergy)	Imipenem	0.7
22	*C. koseri* (3189)	–	–	0.4
23	*C. striatum* (3067)	Benzylpenicillin, cipro*	–	2.4
24	*C. striatum* (3190)	Benzylpenicillin, cipro*, tetracycline	–	No data
25	*K. rhizophila* (3065)	–	–	12
26	*P. mirabilis* (3052)	Gentamicin	Cefuroxime i.v	2.8
27	*P. mirabilis* (3053)	Ampicillin, fosfomycin i.v., piperacillin/tazobactam, trimethoprim/sulfamethoxazole	Cefuroxime i.v	No data
28	*Proteus mirabilis* (3054)	Ampicillin, amoxicillin/clavulanic acid, cefo-taxime, cipro*, trimethoprim/sulfamethoxazole	–	No data
29	*P. penneri* (3055)	Amoxicillin/clavulanic acid, cefotaxime, cipro*, trimethoprim/sulfamethoxazole	Levo**	2.1
30	*P. penneri* (3191)	Amoxicillin/clavulanic acid, cefotaxime, cipro*, trimethoprim/sulfamethoxazole	Levo**	No data
31	*P. monteili* (3069)	Cipro*, levo**	Cefepime, ceftazidime, gentamicin, imipenem, piperacillin/azobactam	2.5
32	*P. aeruginosa* (3070)	–	Imipenem, cefepime, ceftazidime, cipro*, levo**, piperacillin/tazobactam	1.1

PCM numbers are given in parentheses. *S. Staphylococcus,* C. *Corynebacterium.* cipro* ciprofloxacin, levo** levofloxacin.

Antimicrobial photodynamic therapy was performed using 16 µM (5 µg/mL) methylene blue as the photosensitizer and an OLED emitting red light as the light source (radiant exposure energy dose 54 J/cm^2^ at an irradiance 5 mW/cm^2^). Each bacterial strain was measured in three ways: growth control—C, methylene blue effect—MB, and treatment effect—APDT. Experiments were conducted with the use of 96-well plates which enables the culture of all samples at the same time and under the same conditions. Non-irradiated samples were located on the other side of the plate and protected from light by aluminum foil. The basis of the APDT effectiveness assessment is a control sample (C), an untreated bacterial suspension that was kept without photosensitizer and light during APDT treatment where bacterial reduction is equal to zero, so the surviving fraction of bacteria is always equal to 100%. The influence of methylene blue as a photosensitizer was detected by the growth measurement in the group treated with non-activated methylene blue (MB). The last group was bacteria growth in presence of methylene blue and exposed to OLED radiation (APDT). The amount of bacteria remaining after treatment was measured by determining growth curves in a microplate reader as in work by Cheng et al.^23^.

**Figure 2:**
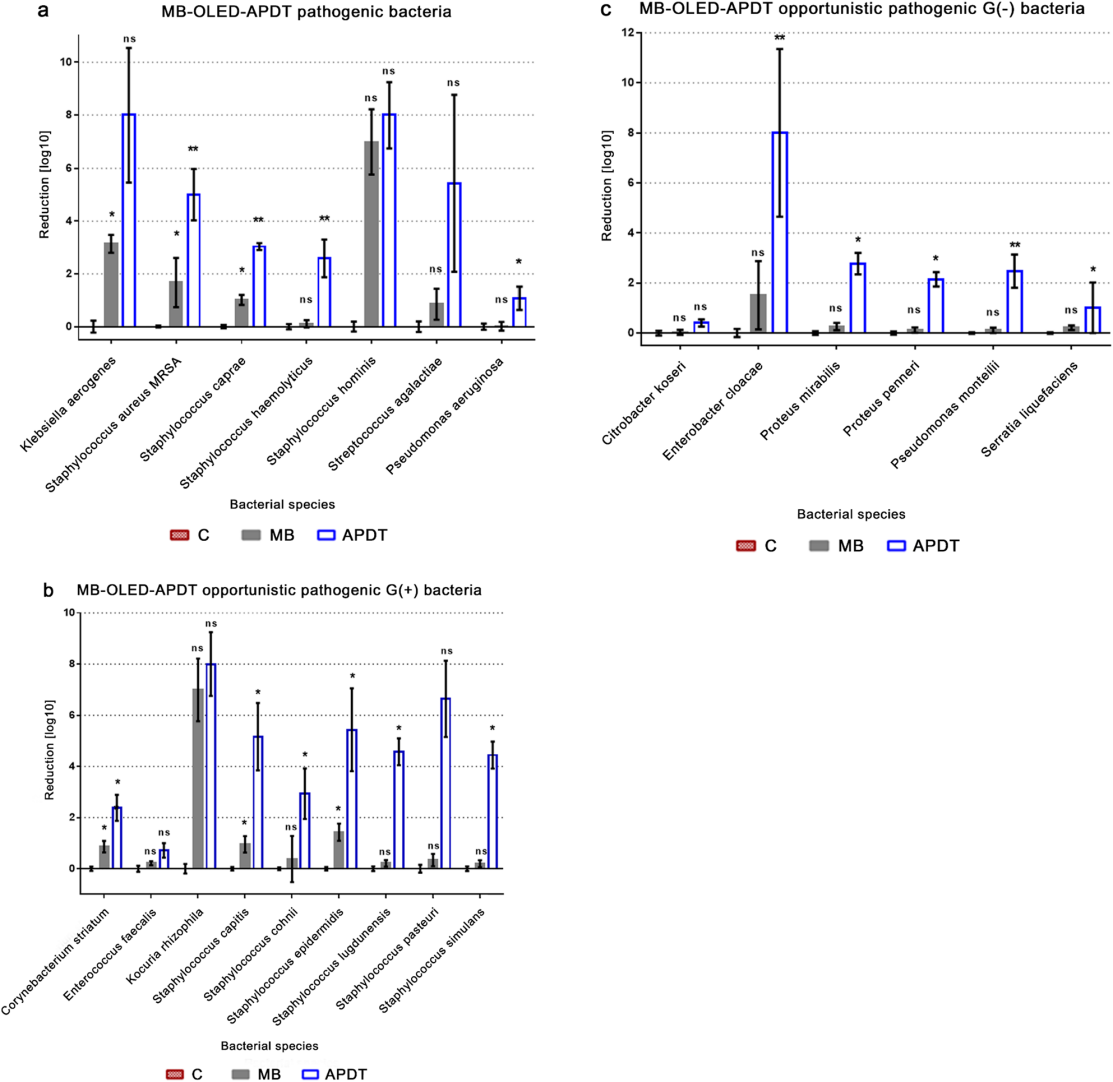
The reduction via OLED-based antimicrobial photodynamic therapy of bacteria isolated from diabetic foot ulcers: (**a**) pathogenic, (**b**) gram-positive opportunistic pathogenic, (**c**) gram-negative opportunistic pathogenic bacteria. The results for each bacterial species are presented in three bars: red (C)—control—non-treated bacterial culture, grey (MB)—bacteria growth in the dark in presence of methylene blue concentration 16 µM (5 µg/mL), and empty blue bars (APDT)—bacteria treated with 16 µM (5 µg/mL) methylene blue and irradiated through 3 h. The results are shown as log reduction of bacteria load to expose the differences between the growth in the MB and APDT samples. Each bar in the graph represents three biological replicates each consisting of three technical replicates (n = 9). Error bars represent standard deviation. T-test with the two tailed distribution and equal variance was performed using Excel 2013, Microsoft Office 2013 (*ns* non-statistical, *p-value < 0.05, **p-value < 0.01). Graphs were made and edited in GraphPad Prism 5.

Since pathogenic bacteria pose a primary danger for infected patients, the effect of photoinactivation was evaluated first on them, and the results are shown in Fig. 2a. In this group, bacterial species responsible for blood and hospital-associated infections including highly antibiotic-resistant bacteria are found. The obtained reduction is between 1.1-log reduction of *Pseudomonas aeruginosa* and > 8-log reduction of *Klebsiella aerogenes*. The reduction of *Staphylococcus aureus* MRSA was 5-log (Fig. 2a). A significant reduction was observed among gram-positive bacteria, especially clinical isolates staphylococci, the most common isolated bacterial genus. For almost all isolated *Staphylococcus ssp.* the obtained reduction was at least equal to 3-log which is equivalent of 99.9% killed bacterial cells. The exception are *Staphylococcus haemolyticus* where reduction is 2.6-log and *Staphylococcus cohnii* with log reduction equal to 2.9-log. The best outcome was obtained for *Staphylococcus hominis* with reduction > 8-log. The toxic effect of methylene blue was observed for all isolated staphylococci but the sensitivity to photosensitizer presence varied between species. The greatest reduction caused by non-activated methylene blue (no light exposition) exhibited *Staphylococcus aureus* (1.6-log reduction). The results for opportunistic pathogenic bacteria are shown in Fig. 2b and c. For gram positive opportunistic bacteria we obtained the best results > 8-log reduction for *Kocuria rhizoph*ila, and for staphylococci, where the results range from 2.9-log (*S. cohnii*) to 6.6-log (*S. pasteuri*). The weakest reduction in this set of bacteria was measured for gram-positive *Enterococus feacalis* 0.7-log. For gram-negative opportunistic pathogens the dominant reduction is around 2-log, however, some species, such as *Enterobacter cloacae,* exhibited unexpectedly high reduction level (> 8-log reduction) and low reduction as *Citrobacter koseri* 0.4-log**.**

## Discussion

As explained in the introduction, the research aim was to evaluate whether antimicrobial photodynamic therapy with an OLED is an effective method for reducing the growth of clinical isolates and thus assess whether it has potential in the treatment of difficult-to-heal ulcers resistant to antibiotic therapies. We identified bacteria colonizing diabetic foot ulcers and tested their susceptibility to antibiotics. Interestingly, among thirty-two isolated bacterial strains, beyond common orthopaedic pathogens such as *Staphylococcus aureus*, *Pseudomonas aeruginosa* and *Enterococcus faecalis*, a number of commensal flora of normal human skin were detected, which could be potentially pathogenic under favourable conditions^24,25^. Their presence in soft tissue infections should not be ignored due to their ability to transfer antibiotic resistance genes to other bacteria, especially pathogens^26,27^.

Our results show that OLED-based antimicrobial photodynamic therapy is effective in eliminating bacteria and has significant potential as a treatment for the wide range bacterial infections found in diabetic foot ulcers. The in vitro bacterial reduction was greater than 3-log (99.9%) for 50% of the identified bacterial species. Most importantly, APDT was effective against highly antibiotic-resistant pathogens such as MRSA, *S. caprae, S. hominis* and *S. agalactiae*, as well as staphylococci broadly present in ulcers and wounds. A similar effect was observed also for majority of gram-negative bacteria. The mechanism of killing bacteria by APDT mechanism is still under consideration. The major hypothesis concerning the susceptibility to APDT relates to differences in bacterial morphology, especially the thickness and composition of the cell wall, the rigidity and porosity of the bacterial membrane, and the presence of an additional outer layer of lipopolysaccharide in gram-negative bacteria may interfere with the effective penetration of the photosensitizer^28^. The main reason for the prevalence of the above hypothesis is that the vast majority of studies on the efficacy of APDT among gram-negative bacteria are limited to two models of gram-negative bacteria, *Escherichia coli* and *Pseudomonas aeruginosa*, characterized by poor reduction. In contrast, according to our and other studies, a significant reduction of pathogenic gram-negative bacteria (*Enterobacter cloacae, Klebsiella aerogenes*) is possible^29^. The exact factors determining the effectiveness of APDT on a single bacterial cell are still under investigation.

As AST results showed, a majority of the isolated bacteria exhibit adaptive antibiotic resistance, and tolerance to antibiotic exposure at low concentration. *Kocuria rhizophila* showed > 8-log reduction showing the great medical potential of APDT which is particularly noteworthy given the prevalence of this bacterium in immunocompromised patients^30^. Factors such as poor circulation lead to a weak immune response in diabetic foot so opportunistic bacteria can cause pathological tissue changes. Antibiotic therapy is usually limited to clinically relevant bacteria, however, such treatment is a high-risk factor for microbial imbalance leading to colonization of life-threatening bacteria such as *Clostridium difficile*, and promoting serious long-term complications^31,32^. This also leads to diagnostic challenges in bacteria identification and makes it difficult to understand the importance of individual strains in the development of tissue pathologies. Taking into account the above factors and the prevalence of antibiotic-resistant bacteria, it is very important to find alternative safe effective treatments for bacterial infections.

The findings demonstrate that APDT provides an effective alternative approach to fighting bacteria in diabetic foot ulcers. It is able to reduce all identified clinical isolates including antibiotic-resistant bacteria. Moreover, there is not a known mechanism of resistance to APDT since defense against singlet oxygen in bacteria is ineffective, and what is more, the mechanisms of the antibiotic resistance do not seem to affect the photodynamic therapy action. Furthermore, the classification of pathogens or species affiliation does not appear to determine the reduction effect, nor does cell wall composition, which establishes affiliation with Gram-positive and Gram-negative bacteria^33^. Another feature of APDT is its local action, attractive for treating topical, superficial bacterial infections such as diabetic ulcers. APDT is also a promising option for patients diagnosed with an allergic reaction to antibiotics, or contraindications to antibiotic therapy, including patients with liver or renal dysfunction or failure. Foremost, photodynamic therapy is medically approved and recommended for treating actinic keratoses, Bowen's disease, basal cell carcinoma, macular degeneration, oesophageal cancer, mouth cancer, and lung cancer^34^.

Our use of OLEDs as the light source for APDT has the capability to make the treatment simple and widely available. An OLED is a compact area light source that can be worn. It, therefore, enables outpatient treatment and even self-care treatment by patients at home. The OLED used here was optimized for potential medical applications. The device design copes with the problem of local heating and high driving voltage concerning other light sources. Furthermore, the red light emitted is considered safe for human cells and capable of penetrating deeper into tissue than shorter wavelengths. A limitation of current light sources for PDT is that they are large, require a hospital visit and are only available in specialized centers. The main disadvantage of commonly used light sources in APDT treatments is the possible discomfort caused by local heating^35^. To overcome this problem we propose the use of a wearable OLED with low irradiance. The unique design and features of OLEDs give this light source great potential for biomedical applications, adhesion to the skin or tissue surface and integration with other medical devices^36–38^.

## Conclusions

Our results show that APDT is a highly effective method for eradicating clinical bacterial strains in vitro including dangerous antibiotic-resistant bacteria as well as opportunistic pathogens. We obtained a reduction by a factor of 1000 (3-log) in the majority of species. Furthermore, we achieved a reduction of at least a factor of 10 in all species. The method is extracorporeal and applied locally thus it poses an attractive alternative for patients in whom antibiotics are not recommended or are insufficient. The use of only one medically approved drug and OLED as a light source, already used in therapeutic wearable devices, makes the therapy safe for patients and has potential to reduce the cost of hospitalization.

APDT is considered to be a promising treatment for bacterial infection in humans, in particular, skin ulcers as in a diabetic foot or other difficult-to-heal wounds. OLED PDT has additional advantage of being feasible for ambulatory treatment. Clinical testing and regulatory approval are needed to translate our results to patients care. Bearing in mind all the above, APDT is considered to be a promising treatment for superficial bacterial infections in humans.

## Materials and methods

### Light source

An organic light-emitting diode was used as a light source in this study. The OLED was designed to be top-emitting, with a microcavity structure, so the emission peak can be tuned to match the absorption of the photosensitizer. The emission layer was Bis(2-methyldibenzo[f,h]quinoxaline)(acetylacetonate) iridium(III) [Ir(MDQ)2(acac)] in a *N*,*N*′-Bis(naphthalen-1-yl)-*N*,*N*′-bis(phenyl)-benzidine (NPB) host, providing a photoluminescence peak of the OLED at 610 nm. By adjusting the thickness of the doped hole transport layer (HTL) the OLED electroluminescent peak can be tuned in the range from 669 to 737 nm. The device is characterized by surface-illumination nature and size-manipulable area. A metal heat sink was used for devices made on glass. The light irradiance was 5 mW/cm^2^ which gives after three hours of irradiation the radiant exposure equal to 54 J/cm^2^. The OLED device was designed with 4 pixel, each pixel can illuminate 12 wells in a 96-well plate. More detail concerning OLED specification is presented in our previous work^23^ (Supplementary Figures).

### Photosensitizer

Methylene blue (MB) was used as a photosensitizer. The stock solution of methylene blue was prepared in PBS with a concentration of 782 µM (250 µg/mL) and sterilized by filtration with the use of a 0.20 µm membrane PTFE filter. The destination final concentration of methylene blue in the bacteria suspension was equal to 16 µM (5 µg/mL).

### Bacterial swabs

Bacteria were obtained from Dobrzyńska Medical Center WZSOZ (Wrocław, Poland) from patients with clinical evidence of soft tissue infection in foot. After the wound has been cleansed and debrided the clinical specimens were collected from ten diabetic foot ulcers via the Levine technique by using Amies Agar Gel Transport Swab (Deltalab, Spain). The samples were anonymized and numbered in the order they were taken. The entire procedure was performed by experienced medical staff.

### Bacteria species identification

The mixed bacterial culture from single swab was lead on the four agar plates with different media, including MacConkey agar, blood agar, brain heart infusion (BHI) agar and lysogeny broth (LB) agar. The culture was incubated 24 h and single colonies were streaked on fresh plates to obtain pure bacterial culture. The microbial identification and taxonomical classification was based on matrix-assisted laser desorption/ionization time-of-flight mass spectrometry (MALDI-TOF MS) according to the procedure described by Seng et al.^39^ Further analysis was conducted with MALDI BioTyper 3.1 software with the use of Bruker Daltonics Database BDAL (Supplementary Figures).

### Antibiotic susceptibility testing (AST)

To determine the antibiotic susceptibility among isolated bacteria, all species were tested commercially by Medical Microbiological Laboratory DIAGNOSTYKA according to the procedure IB/LAB/1773 I version from 2018-10-01 by culture method confirmed by mass spectrometry, antimicrobial susceptibility was determined by the broth microdilution method using the MicroScan WalkAway diagnostic microbiology system.

### Antimicrobial photodynamic therapy evaluation

The evaluation of the efficiency of photodynamic inactivation was performed on planktonic bacteria in vitro. Overnight culture of chosen identified bacteria species was standardized to an optical density (OD) equal to 0.001 then three groups were prepared: growth control (C) containing bacteria cells suspension without photosensitizer (no PS, non-irradiated), photosensitizer control (MB) illustrating the influence of the photosensitizer in the dark (with PS, non-irradiated) and photoinactivated group (APDT) containing methylene blue and expose to OLED radiation (with PS, irradiated). The final methylene blue concentration in the bacterial suspension was 5 µg/mL. All groups were placed at 96-well plate (250 µL per well) and incubated at 37 °C for 3 h with or without light. OLED was placed directly on the surface of the plate (circa 4 mm distance) and during 3-h-irradiation it delivered 54 J/cm^2^ at the irradiance 5 mW/cm^2^. The plate was divided into an area of illumination and dark area to ensure uniformity of conditions. The groups were prepared using one overnight bacterial suspension which poses one biological repetition. In addition, each group has been split into three wells which provided three technical repetitions (n = 3). To obtain statistical data, three biological replicates were performed resulting in a total of nine technical replicates per result (n = 9). After APDT process plate was centrifuged at 4750×*g*, 5 min and supernatant was replaced by fresh medium.

### Bacteria growth evaluation

Measurements were conducted as previously described in Lian et al.^23^ The growth measurement of bacteria was carried out in CLARIOstar® Microplate Reader (BMG LABTECH). After medium replacement plate was placed in the reader and incubated at 37 °C in the dark with continuous double orbital shaking (300 rpm) for at least 20 h. The absorbance at 450 nm was measured every 15 min which enables to generate accurate growth curves. The inhibition effect of methylene blue or APDT translates directly into much slower increase the absorbance over time and lower absorbance in the end. The method used to compare obtained results was the high-throughput Start-Growth-Time (SGT).

### Ethics declarations

The procedures and protocols were carried out as part of the project: “Application of antimicrobial photodynamic inactivation in the control of bacterial skin infections”. This project was approved by the Scientific Council of the Institute of Immunology for year 2020 and extended for 2021 and 2022. (Resolution No. 10/199/2019 of the Scientific Council of the Ludwik Hirszfeld Institute of Immunology and Experimental Therapy of the Polish Academy of Sciences in Wrocław, dated 12 December 2019, Resolution No. 6/e-204 /2020 dated 10 December 2020, and Resolution No. 19/e-208/2020 dated 9 December 2021).

All methods used in the study were performed in accordance with the Declaration of Helsinki. Animals were not used in the study.

The human material used in the experiments was collected from the patients after informing them about the procedure and the purpose of the study and after obtaining their written consent.

### Supplementary Information

Supplementary Figures.

## Data Availability

The datasets used and/or analyzed during the current study are available from the corresponding author upon reasonable request.
