# Supplementary material for: Treatment of antibiotic-resistant bacteria colonizing diabetic foot ulcers by OLED induced antimicrobial photodynamic therapy
Source: Sci Rep. 2023 Aug 28;13:14087. doi: 10.1038/s41598-023-39363-4 (PMC10462621; doi:10.1038/s41598-023-39363-4)
Supplement: Supplementary file 1 — Supplementary Figures. [file 41598_2023_39363_MOESM1_ESM.pdf]

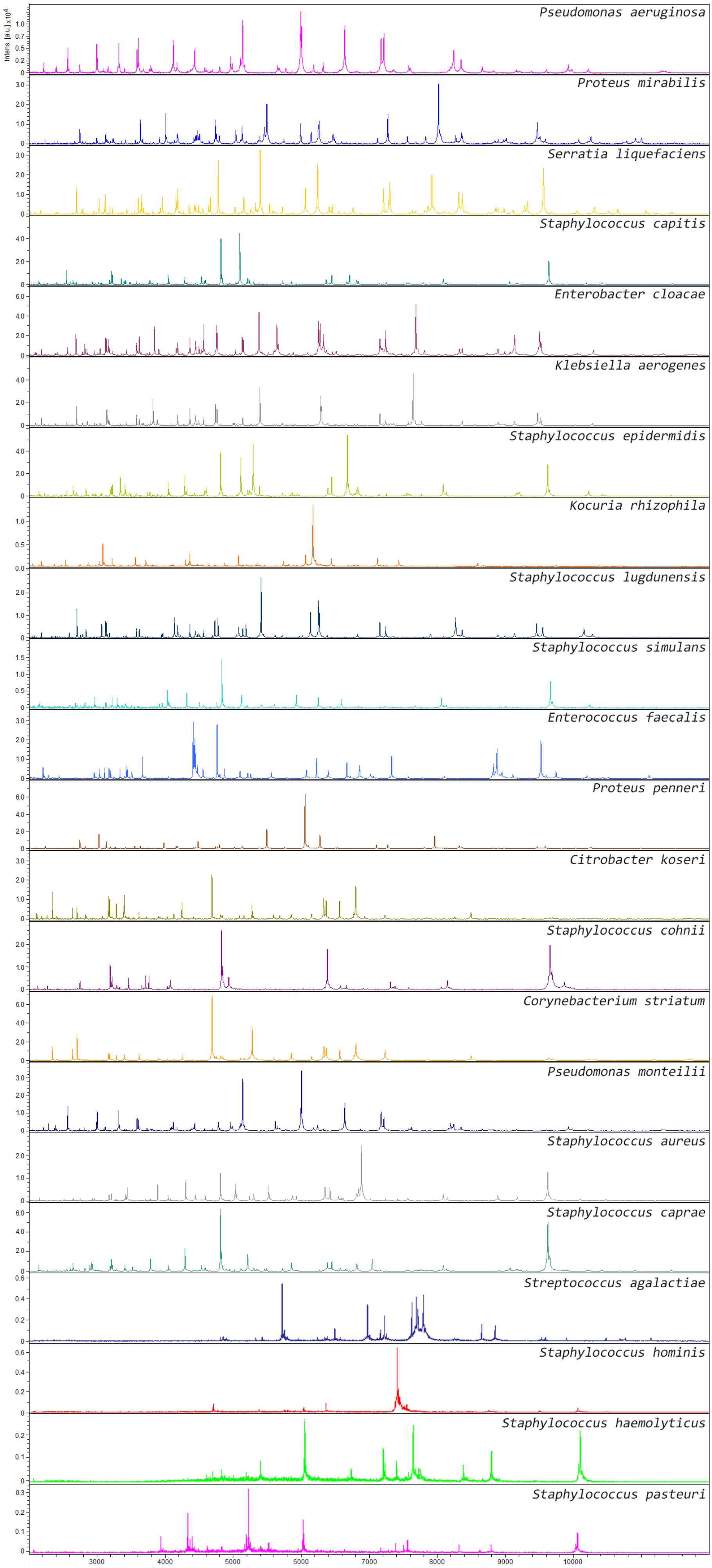

Intens. [a.u.] x10^4
Pseudomonas aeruginosa
Proteus mirabilis
Serratia liquefaciens
Staphylococcus capitis
Enterobacter cloacae
Klebsiella aerogenes
Staphylococcus epidermidis
Kocuria rhizophila
Staphylococcus lugdunensis
Staphylococcus simulans
Enterococcus faecalis
Proteus penneri
Citrobacter koseri
Staphylococcus cohnii
Corynebacterium striatum
Pseudomonas monteilii
Staphylococcus aureus
Staphylococcus caprae
Streptococcus agalactiae
Staphylococcus hominis
Staphylococcus haemolyticus
Staphylococcus pasteuri
3000
4000
5000
6000
7000
8000
9000
10000

Figure S1

The MALDI-TOF MS enabled to obtain unique for each bacterial species the protein fingerprints which were further analysed with the use of MALDI Biotyper software (Bruker Daltonics, Germany). The obtained spectra were compared with the spectra collection - Bruker Daltonics database (BDAL). Matches were assessment by Meaning of Score Value and values located in the range of the highest score were accepted. In case of uncertain outcome, the entire analysis was conducted anew. The strains identification is confirmed by the independent analysis conducted by Polish Collection of Microorganisms (PCM).

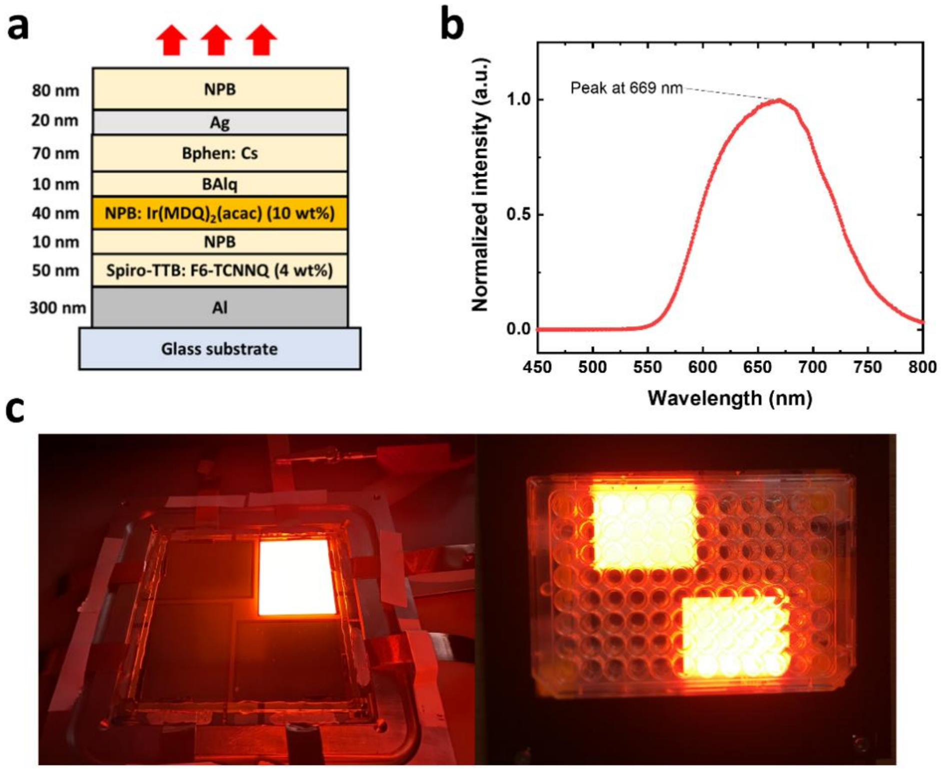

Figure S2

OLED details for antimicrobial PDT. a) Top-emitting microcavity OLED with doped transport layer device structure. b) Normalized OLED spectrum with an emission peak at 669 nm. c) 4-pixel OLED designed for 96 well-plate illumination.
